# A Systematic Literature Search on Psychological First Aid: Lack of Evidence to Develop Guidelines

**DOI:** 10.1371/journal.pone.0114714

**Published:** 2014-12-12

**Authors:** Tessa Dieltjens, Inge Moonens, Koen Van Praet, Emmy De Buck, Philippe Vandekerckhove

**Affiliations:** 1 Centre for Evidence-based Practice, Belgian Red Cross-Flanders , Mechelen, Belgium; 2 Psychosocial Intervention Service, Belgian Red Cross-Flanders, Mechelen, Belgium; 3 Department of Public Health and Primary Care, Faculty of Medicine, Catholic University of Leuven, Leuven, Belgium; 4 Faculty of Medicine, University of Ghent, Ghent, Belgium; National Center of Neurology and Psychiatry, Japan

## Abstract

**Background:**

Providing psychological first aid (PFA) is generally considered to be an important element in preliminary care of disaster victims. Using the best available scientific basis for courses and educational materials, the Belgian Red Cross-Flanders wants to ensure that its volunteers are trained in the best way possible.

**Objective:**

To identify effective PFA practices, by systematically reviewing the evidence in existing guidelines, systematic reviews and individual studies.

**Methods:**

Systematic literature searches in five bibliographic databases (MEDLINE, PsycINFO, The Cochrane Library, PILOTS and G-I-N) were conducted from inception to July 2013.

**Results:**

Five practice guidelines were included which were found to vary in the development process (AGREE II score 20–53%) and evidence base used. None of them provides solid evidence concerning the effectiveness of PFA practices. Additionally, two systematic reviews of PFA were found, both noting a lack of studies on PFA. A complementary search for individual studies, using a more sensitive search strategy, identified 11 237 references of which 102 were included for further full-text examination, none of which ultimately provides solid evidence concerning the effectiveness of PFA practices.

**Conclusion:**

The scientific literature on psychological first aid available to date, does not provide any evidence about the effectiveness of PFA interventions. Currently it is impossible to make evidence-based guidelines about which practices in psychosocial support are most effective to help disaster and trauma victims.

## Introduction

In the first few moments and hours after a disaster, survivors may have medical, material, social, and emotional needs. After the traditional steps to guarantee physical safety, it became common practice to also offer immediate psychosocial support [1], [2]. A contemporary definition of psychosocial support is given by the International Federation Reference Centre for Psychosocial Support of the Red Cross and Red Crescent Societies (2011) as “a process of facilitating resilience within individuals, families and communities” [3]. This is based on the idea that people can rely on their own strengths to recover from the impact of a disaster or an adversity. Psychosocial support arose from the merger of social and psychological support. Social support lies at the heart of humanitarian aid organizations ever since their founding in the second half of the 19th century, fulfilling practical and social needs (e.g. reestablishing contacts with family members). Early psychological support following critical incidents was originally developed to support military personnel [4]. After the recognition of post-traumatic stress disorder (PTSD) as a psychiatric disorder in 1980 [5], the idea to prevent psycho-trauma entered the work of humanitarian aid agencies from the beginning of the 1990s [6]. However, on the field, trauma focused interventions proved to be ineffective and even harmful [7]. Safer interventions where those that addressed the needs of the affected [8]. Subsequently the idea of early psychological interventions merged with the social approach [9], leading to the concept of psychosocial support. A wide range of interventions were developed to provide psychosocial support. Today, one such intervention strategy is psychological first aid (PFA) [10]. PFA is defined by the World Health Organization (WHO) as “a humane, supportive response to a fellow human being who is suffering and who may need support” [11]. It includes interventions such as listening, comforting, helping people to connect with others and providing information and practical support to address basic needs [11], [12]. These interventions are consistent with the guidelines of Hobfoll et al. [13] and center on five key principles: safety, connectedness, self and collective efficacy, calm and hope, that together in essence ease the transition to normality [13]. This implies that the practice of PFA is not restricted to mental health professionals but could also be delivered by lay people.

Training people in PFA improves their confidence in applying it [14]. The Belgian Red Cross-Flanders (BRC) offers basic 3 hours PFA courses to lay people (aiming to prepare them for standardized care), and a 28 hours course to health professionals (aiming to prepare them for individualised care). These trainings, developed by the BRC-Psychosocial Intervention Service, are based on several published international guidelines for PFA [3], [9], [11], [13], [15]. The use of evidence-based guidelines to develop practice manuals or trainings is becoming the gold standard for organizations such as the World Health Organization [16] and the Red Cross [17], [18]. When using the evidence-based practice methodology the best available objective evidence is integrated with expert opinion and preferences from the target population in order to provide high-quality guidelines. Systematic literature searches are considered the cornerstone of evidence-based practice and aim to collect all well-designed research by performing a computerized search of large reference sources [19].

In this paper, a search for evidence was performed to develop an evidence-based guideline on PFA, aimed at improving our own trainings. We started by critically evaluating the evidence base included in existing guidelines and systematic reviews on PFA. We then performed an extensive literature search, applying a more sensitive approach compared to previous searches, to identify studies on the effectiveness of PFA.

## Materials and Methods

### Key Definitions

Considering the broad and unclear terminology used within the PFA domain, we will first set out some key definitions relevant to this paper, in order to be transparent in our own terminology.

**Psychological first aid**: We conceive PFA as an intervention approach aimed at helping people deal with the experience and the consequences of a disaster or adversity. Our practice is strongly based on the five principles of Hobfoll [13] meaning that the main purpose of PFA is to install feelings of safety, calmness, self- and community efficacy, connectedness and hope.

**Immediate aftermath of a disaster**: a couple of hours until 7 days after the event

**Mental health professionals**: people with a psychological/social degree

**Laypeople**: People without any previous training in the field of psychological or social support

### Identification of existing guidelines and systematic reviews

A primary search (until July 2013) was performed to identify PFA guidelines (using the G-I-N database and MEDLINE) and to identify systematic reviews with a focus on PFA (using The Cochrane Library and MEDLINE) (S1 Appendix). Guidelines were included if they reported the development process (e.g. use of systematic literature search, expert meeting, consensus method used). Systematic reviews had to fulfill the PRISMA criteria in order to be included [20]. Hand searching was done to find additional systematic reviews. Reference lists of included publications were searched for relevant citations.

### Search for individual studies

The search for individual studies focused on the following question: “In people affected by a disaster or trauma do certain PFA interventions, promote safety, connectedness, self and collective efficacy, calm and hope?”. Search strategies were developed by the BRC Centre for Evidence-Based Practice in cooperation with the BRC Psychosocial Intervention Service. Electronic searches of literature were conducted in the following databases: The Cochrane Library, MEDLINE (PubMed interface), PsycINFO, PILOTS (a database specialized in PTSD literature), from the date of inception until July 2013. Highly sensitive search strategies have been used in order to be as complete as possible in the search for scientific literature. Search terms such as ‘PFA’ and its synonyms were extended with terms referring to the five essential elements individually (safety, calm, self and collective efficacy, connectedness and hope) as described by Hobfoll et al. [13] (S1 Appendix) to broaden the search and to increase search results. Additionally, references from relevant studies, reviews and guidelines, published on the same topic, were screened for supplementary articles. The initial study selection was performed by one author (TD). Full text evaluation was done by two authors (TD and IM). Disagreements between reviewers were resolved through discussion involving all authors (methodological experts and content experts).

Articles were included when containing the following characteristics: (1) population: victims of a disaster or traumatic event; not diagnosed or referred by a health professional; (2) intervention: community-based interventions; by laypeople, first responders, health care professionals, the victim himself; taking place the first few hours/days of a disaster, single interventions; feasible for laypersons (3) measuring mental health parameters (resilience, efficacy, empowerment, stress, coping, functioning, engagement, etc.) or physiological parameters (blood pressure or heart rate). Studies must have a controlled experimental or observational study design in order to be included. Studies on individual treatment or one-to-one sessions, therapeutic interventions after a diagnosis by a health care professional; medical interventions, long term interventions and psychological debriefing were excluded.

### Data analysis

The specific data extracted were (1) methodological development and content for guidelines and systematic reviews; (2) details on the study type, population, intervention and outcomes for individual studies. The Appraisal of Guidelines for Research and Evaluation II (AGREE II) instrument [21] was used to assess the quality of the guideline development processes, particularly for rigour of development. In this tool, a seven point scale (Strongly Disagree ( = 1) to Strongly Agree ( = 7)) was used to measure the extent to which each of the criteria has been fulfilled by the guideline. To score the rigour of development, eight elements were evaluated: search methodology, selection criteria, evidence quality assessment, consideration of health benefits, side effects, and risks, link between recommendation and evidence, review by external experts, and update information. The guidelines were evaluated by two assessors (TD and HVR).

## Results

### Quality of existing guidelines and systematic reviews concerning PFA

A total of five published practice guidelines were identified by the search strategy [13], [15], [22]–[24]. Three were developed in Europe, one in the USA and one in Australia, and all were published between 2007 and 2012. Funding for the development of the guidelines was provided by governmental institutions (Bisson (TENTS), EU funded; Hobfoll, US Government funded; Vymetal (EUTOPA), EU funded; Kelly, Australian Government funded; Te Brake, Dutch Government funded). The EUTOPA-guidelines [24] are based on the Dutch guidelines from Te Brake et al. [23], but were adapted to a European context. Results of the methodological quality assessment by AGREE II are shown in Table 1. The quality with respect to “Rigour of Development” was variable. Four guidelines explicitly mentioned their method of development [15], [22]–[24]. The guidelines by Bisson [15], Te Brake [23] and EUTOPA [24] have the highest rigour of development. All three explicitly stated their strategy to search for evidence on effective interventions used in PFA, prior to the consultation of experts (Table 1). The methodology section of Kelly et al. [22] reported a literature search, with the difference that the search was not focused on effective interventions, but rather on gathering information on every possible action done after a traumatic event. Hobfoll et al. [13] used a different approach, including indirect evidence from related fields, not collected by a systematic search. The criteria for selecting the evidence were described only in the TENTS guidelines [15]. Methods for formulating recommendations were clearly described in the TENTS guidelines and Kelly et al., both using the Delphi consensus methodology. We found no information of external review prior to the publication for any of the evaluated guidelines. A statement about the procedure for updating the guideline was only provided in the EUTOPA guidelines. All guidelines mention the lack of randomized controlled trials to study interventions in disaster settings. None of the studies included in the guidelines did fulfill our predefined inclusion criteria. S2 Appendix contains a list of these studies with the main reason for exclusion.

**Table 1:** Overview of existing guidelines.

	Guideline	Topic	Recommendations based on	Search	Search strategy *[number of references screened]*	Included studies concerning PFA interventions	Guideline development	AGREE II score* (methodology) %
Bisson 2010	TENTS Guidelines (The European Network for Traumatic Stress)	Immediate response after natural and other disasters	a systematic review	MEDLINE, Embase, PsycINFO, HMIC, INSPEC, Journals@Ovid, British Nursing Index Archive, AMED, All EBM Reviews; World wide web	(Disaster OR "major traumatic event" OR catastrophe OR earthquake OR flood OR terrorism OR tsunami OR hurricane OR fire OR tornado) AND ("mental health" OR PTSD OR "traumatic stress" OR psychological OR social) AND ("clinical trial" OR meta-analysis OR "randomized clinical trial" OR "phase I" OR "phase II" OR "phase III" OR "phase IV" OR "comparative study" OR "controlled clinical trial" OR cohort OR longitudinal) *[650 references]*	Boscarino 2006; Hodgkinson 1993; Najarian 2001; Thienkrua 2006; Van Griensven 2006; Wolmer 2005	Delphi process; guidelines are result of consensus of expert opinion guided by the evidence available; European context; Experts are TENTS partners with expertise in the trauma field.	53
Kelly 2010	Mental Health First Aid Guidelines	Appropriate early mental support after a traumatic event by laypersons	a literature search, NOT a systematic review	PsycINFO, MEDLINE; to identify first aid actions, no quality assessment; World wide web (Google); Amazon books; Pamphlets from aid organisations; guidelines for professionals, training course	trauma*; 'traumatic event' (Google); 'traum' OR 'posttraumatic stress disorder' (Amazon) *[not applicable]*	NA	Delphi process; guidelines are result of consensus of expert opinion; the panel included: Professionals, consumers, carers from ENG speaking countries (UK, NZ, Australia, Canada, US); the literature was used to develop statements in the questionnaire for the Delphi process.	35
Hobfoll 2007	Mid-term Mass Trauma Guidelines	Interventions during the immediate and the mid–term post mass trauma phases	studies from related fields of research (extrapolation)	NA	NA	NA	Recommendations based on consensus by a worldwide panel of experts on the study and treatment of those exposed to disaster and mass violence.	20
Te Brake 2009	National Guidelines on Psychosocial Interventions	Early psychosocial interventions after disasters, terrorism and other shocking events	a systematic review	NGC, GIN, Cochrane, MEDLINE, PsycINFO, Pilots	‘disasters’, ‘terrorism’, ‘acute posttraumatic stress’, ‘acute psychological interventions’, ‘crisis care’, ‘brief interventions’, ‘debriefing’*[not mentioned]*	a) Supportive context: no studies b) Psychosocial interventions: Information: no studies; Psycho-education:[Ehlers 2003; Turpin 2005; Sijbrandij 2007; Everly 2002; Roberts 2006]; PFA and CISM: [Everly 2002; Roberts 2006]	Recommendations based on available evidence from literature and additional considerations from the panel; The panel included 21 experts (dutch healthcare professionals).	42
Vymetal 2011	EUTOPA Guidelines (European Guidelines for Target Group-Oriented Psychosocial Aftercare)	Optimal psychosocial support and care after experiencing disasters and shocking events	a systematic review	NGC, GIN, Cochrane, MEDLINE, PsycINFO, Pilots	‘disasters’, ‘terrorism’, ‘acute posttraumatic stress’, ‘acute psychological interventions’, ‘crisis care’, ‘brief interventions’, ‘debriefing’ *[not mentioned]*	a) Supportive context: no studies b) Psychosocial interventions: Information: no studies; Psycho-education:[Rose 1999; Ehlers 2003; Turpin 2005; Sijbrandij 2007];	Recommendations based on scientific studies, along with considerations of the European and international expert panel and knowledge of the experiences of those affected.	51

*average of the scores of 2 independent reviewers, rounded to nearest whole number. NA: not applicable.

Next to the 5 guidelines, the search identified two systematic reviews: one developed by Fox et al. (from 1990 to September 2010) supported by the American Red Cross and another by Bisson & Lewis (from inception of the database to 2009) commissioned by the World Health Organization [25], [26]. The search strategies of these systematic reviews were limited to the phrase “psychological first aid” or “PFA”, resulting in respectively 275 and 516 references for screening (Table 2). Similarly to the guidelines, controlled studies on PFA were not identified in any of the two systematic reviews and the authors from both papers call for more research in the PFA field.

**Table 2:** Overview of existing systematic reviews.

	Topic	Search	Search strategy	Number of screened references*	Included studies
Bisson 2009	The effectiveness of early interventions to prevent PTSD, psychiatric disorders following extreme stressors	MEDLINE, Embase, PsycINFO, HMIC, British Nursing Index Archive, AMED, ASSIA, CINAHL, Cochrane, ISI Science Citation Index, ISI Social Sciences Citation Index, IBSS, PILOTS, Sociological Abstracts	'psychological first aid' OR 'PFA'	516	No studies identified
Fox 2012	The effectiveness of psychological first aid as a disaster intervention tool	Cochrane, MEDLINE, PsycINFO, PsycArticles, Pilots (between 2008–2010)	'psychological first aid'	275	No studies identified
This paper	The effectiveness of early PFA interventions	GIN, Cochrane, MEDLINE, PsycINFO, PILOTS (from inception - 2013)	Very sensitive search strategy (See S1 Appendix)	10097	No studies identified

*after removal of duplicates.

Analysis of the search strategies, included in the guidelines and systematic reviews, revealed rather specific searches yielding less than thousand references for screening. To further explore the presence or absence of studies on PFA, we built a more sensitive search strategy and conducted our own systematic literature search.

### Systematic literature search

**Figure 1:**
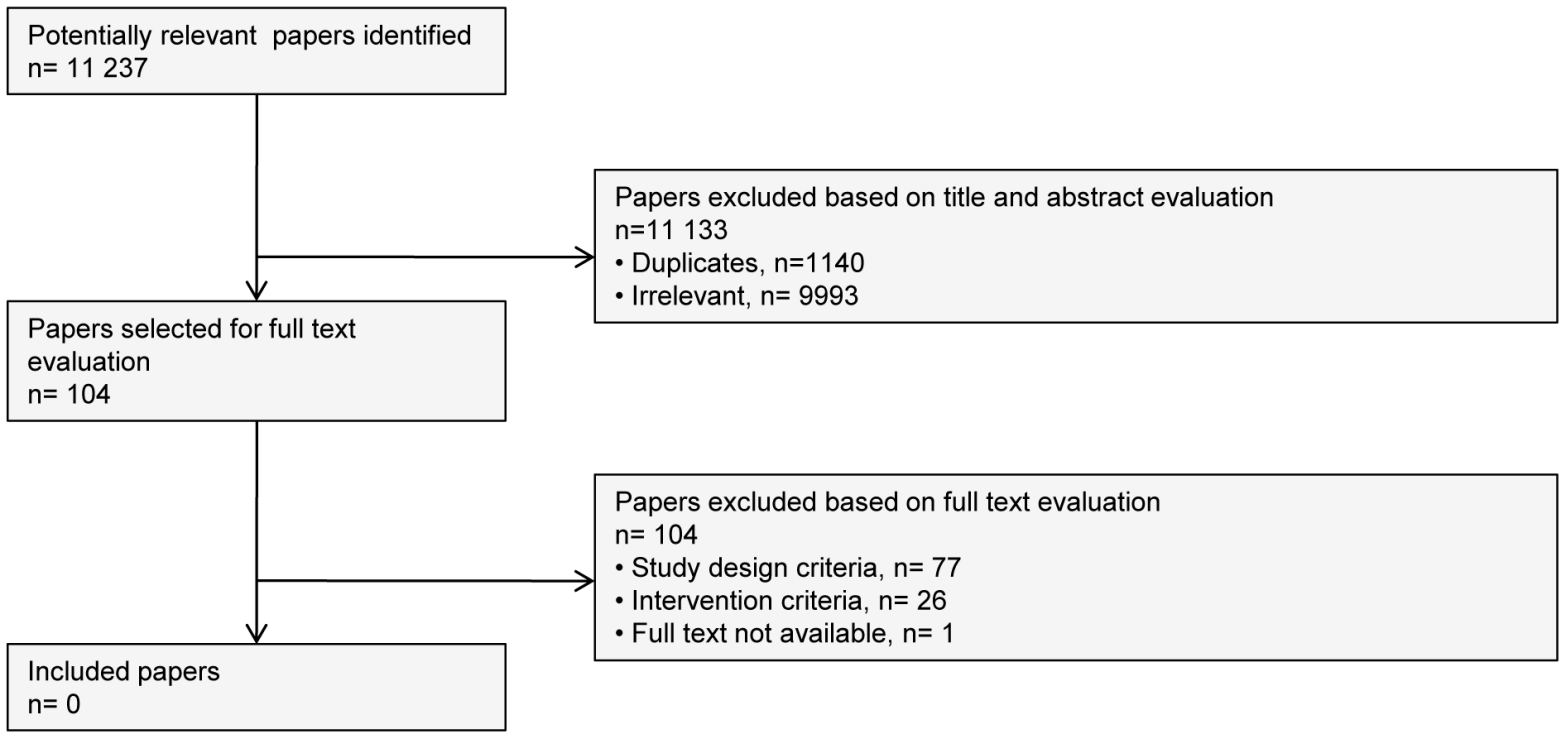
Flow diagram for selecting studies for review.

The highly sensitive search identified 11 237 titles, with 10 097 remaining after deduplication. A total of 104 records were defined as potentially eligible based on title and abstract. Fig. 1 provides information on the number of studies identified, selected or excluded and the main reasons for exclusion. One study was not available in our libraries [27]. Most papers were excluded due to their study design (73%) either because a control group was missing or no intervention was studied, or because it conceived a narrative review, opinion or editorial with no relevant primary data (S2 Appendix). In addition, 25% of the studies did not investigate an intervention of interest to this review. Two studies were excluded after discussion due to disagreement. Declercq et al. was excluded because the researchers focused on PTSD [28]; Verschuur et al. investigated a combination of interventions (medical exam and communication about health consequences of exposure to an avian disaster), with the former not relevant to our research [29]. In conclusion, we could not identify any study that scientifically examined the effects of the various psychosocial measures to support disaster victims.

## Discussion

At the base of evidence-based guideline development lies the use of systematic literature reviews to assess existing research regarding the effectiveness of interventions. The aim of the present article was to more sensitively investigate the evidence supporting PFA guidelines and materials. A detailed analysis of 5 available guidelines unravels that they refer to different references or sources as a base for their recommendations [13], [15], [22]–[24]. This can be due to different methodological development approaches, ranging from an evidence-based methodology to a consensus-reaching approach. Remarkably, none of them provide any evidence concerning the effectiveness of PFA interventions. In addition, two systematic reviews, using specific search strategies with a focus on PFA, also document the absence of intervention studies [25], [26]. In order to make sure that no evidence was missed, a more sensitive search strategy was designed. Despite the higher sensitivity of the search, as compared to the searches in existing guidelines and systematic reviews, no studies could be identified concerning the effectiveness of PFA interventions. Reliable scientific evidence to prove the benefits or reveal the risks of current PFA practices is therefore lacking in the field of PFA.

The lack of research evaluating non-therapeutic PFA interventions, might find its origin on different levels. First of all, scientific evidence in the field of prehospital care is scarce in general, and the available studies often use flawed methods leading to evidence of a very low quality [30], [31]. Secondly, studies during the aftermath of a disaster or adversity are considered difficult to perform. Practical issues such as unpredictability of timing and context are some of the challenges typically invoked, as are ethical issues claiming that research could impede the capacity to respond in this critical time of need [32]–[34]. The third explanation for the evidence gap can be found in the particular domain of PFA, as PFA is a multifactorial intervention based on five key principles as outlined by Hobfoll et al. [13] PFA interventions therefore can take on many different forms depending on the contexts and cultures in which disasters or adversities occur [2], [35]. Each of these interventions should be evaluated separately in experimental studies to gain knowledge on their effectiveness. Finally, in the domain of behavioral sciences, resistance by certain professionals towards evidence-based practice and a lack of uniform definitions and terminology, might contribute to the lack of evidence. A negative attitude about evidence and evidence-based practice can be due to a lack of training and misconceptions regarding the concept of evidence-based practice [36], [37]. However, steps are taken in the right direction: the American Psychological Association (APA) for example stated an explicit commitment to the use of evidence-based practice within all aspects of the profession [38], [39]. Moreover, the lack of uniform terminology in the field of psychology leads, in the case of PFA, to several definitions, frameworks, and interventions [1], [12], [40], [41]. In order to make evidence-based research for PFA easier, an international consensus should be reached on the definitions of these concepts. The European Red Cross/Red Crescent Network for Psychosocial Support (ENPS) already set definitions for PFA, psychosocial support and psychoeducation in its annual forum of 2013.

Evidence-based approaches for psychosocial support after adversities are increasingly being demanded [42]–[45]. A needs assessment study, performed by the Evidence Aid initiative, identified that experts consider evidence on the effects of mental health and psychosocial support interventions as one of the top 30 priorities in disaster research [46], [47]. Several initiatives are taken to bridge the gap between research, guidelines and practice [48], [49]. However, without a reliable evidence base of well-performed studies all guidelines will be expert rather than evidence-based. Therefore, research efforts are urgently needed to demonstrate which interventions are beneficial or harmful to guarantee the most effective psychological first aid for disaster affected populations.

### Strengths and limitations

The limitations of this systematic literature search are important to recognize. Even though a very sensitive search strategy was adopted, there is no absolute guarantee of not having missed relevant articles as much of the literature is not well-indexed in the bibliographic databases. Secondly, the systematic search for papers was restricted to five databases, most relevant to our topic (G-I-N, MEDLINE, PsycINFO, The Cochrane Library and PILOTS). Thirdly, rigorous selection criteria were used to ensure that only highly relevant evidence was retrieved, focusing on non-therapeutic PFA interventions within the first seven days following a disaster which might explain why no studies could be included in the systematic review. The lack of evidence for PFA interventions obviously does not prove evidence of absence of a useful effect of PFA, but it does reveal the need for future studies on the effectiveness of early PFA interventions to support the relatively new concept of PFA.

### Conclusion

Although PFA is considered to be an important approach for disaster-affected populations, there is a complete lack of high-quality experimental and observational studies on the effectiveness of PFA in the immediate aftermath of a disaster. Consequently, research is needed to determine the most effective, efficient, and acceptable interventions before evidence-based PFA guidelines on how to train laypeople and professionals can be developed.

## Supporting Information

S1 Checklist **PRISMA Checklist.**
(DOC)

S1 Appendix **Search strategies.**
(DOC)

S2 Appendix **Excluded studies after full text evaluation.**
(DOC)
