# Supplementary material for: A Systematic Literature Search on Psychological First Aid: Lack of Evidence to Develop Guidelines
Source: PLoS One. 2014 Dec 12;9(12):e114714. doi: 10.1371/journal.pone.0114714 (PMC4264843; doi:10.1371/journal.pone.0114714)
Supplement: S1 Appendix — Search strategies. (DOC) [file pone.0114714.s002.doc]

**APPENDIX S1**. Search strategies

**GIN - guidelines**

- Psychological first aid
- Psychosocial first aid
- Crisis management
- Critical incident
- Posttraumatic

**MEDLINE (PubMed interface) - guidelines & systematic reviews**

((((“Practice Guidelines as Topic”[Mesh] OR “Guidelines as Topic”[Mesh] OR guideline*[TIAB] OR “systematic review”[TIAB] OR “review”[Mesh])) AND (“systematic search”[TIAB] OR “evidence-informed”[TIAB]) OR “consensus-based”[TIAB] OR “evidence-based”[TIAB] OR “Scientific literature”[TIAB] OR “Evidence-Based Nursing”[Mesh] OR “literature search”[TIAB] OR MEDLINE [TIAB] OR PsycInfo [TIAB]) AND (“Disasters”[Mesh] OR disaster*[TIAB] OR traumatic event*[TIAB])) AND “Social Support”[Mesh] OR “psychological first aid”[TIAB]

**The Cochrane Library - systematic reviews**

((Disaster*):ti,ab,kw or (“crisis”):ti,ab,kw or (“crises”):ti,ab,kw or (“emergency”):ti,ab,kw or (“emergencies”):ti,ab,kw or [mh “Disasters ”] OR (traumatic NEXT event*):ti,ab,kw ) AND ([mh “Social Support”] OR (“psychological first aid”):ti,ab,kw OR (“psychosocial support”):ti,ab,kw OR (“mental health”):ti,ab,kw OR (“behaviour health”):ti,ab,kw)

**MEDLINE (PubMed interface) - experimental and observational studies**

1. Disaster[TIAB] OR disasters[TIAB] OR Crisis[TIAB] OR Crises[TIAB] OR Emergency[TIAB] OR emergencies[TIAB] OR ((traumatic[TIAB] OR serious[TIAB] OR destructive[TIAB] OR mass[TIAB] OR stressing[TIAB]OR critical[TIAB]) AND (Event[TIAB] OR events[TIAB] OR incident[TIAB] OR incidents[TIAB] OR violence[TIAB] OR trauma[TIAB])) OR "Disasters"[Mesh] OR "Emergencies"[Mesh] OR "Droughts"[Mesh] OR terrorism[TIAB] OR drought*[TIAB] OR "Cyclonic Storms"[Mesh] OR hurricane*[TIAB] OR "Floods"[Mesh] OR flood*[TIAB] OR "Fires"[Mesh] OR forest fire*[TIAB] OR "Geological Processes"[Mesh] OR "Radioactive Hazard Release"[Mesh] OR tidal wave*[TIAB] OR volcanic eruption*[TIAB] OR tsunami*[TIAB] OR landslide*[TIAB] OR avalanche*[TIAB] OR earthquake*[TIAB] OR Storm*[TIAB] OR wildfire*[TIAB] OR Snowstorm*[TIAB] OR heat wave*[TIAB] OR cold wave*[TIAB] OR land fire*[TIAB] OR Sandstorm*[TIAB] OR "Terrorism"[Mesh] OR massacre [TIAB] OR bomb*[TIAB] OR Evacuation[TIAB] OR "Accidents, Traffic"[Mesh]

2. Provider*[TIAB] OR responder*[TIAB] OR worker*[TIAB] OR helper*[TIAB] OR professional*[TIAB] OR volunteer*[TIAB] OR nurse*[TIAB] OR EMT[TIAB] OR "Emergency medical technician"[TIAB] OR "Caregivers"[Mesh] OR laypeople [TIAB] OR layperson*[TIAB] OR "Survivors"[Mesh] OR evacuees[TIAB]

3. ((Psychosocial[TIAB] OR Psychological[TIAB] OR social[TIAB] OR "mental health"[TIAB]) AND ("first aid"[TIAB] OR care[TIAB] OR support[TIAB] OR assist*[TIAB] OR management[TIAB])) OR Inform*[TIAB] OR Comforting[TIAB] OR communicat*[TIAB] OR conversation[TIAB] OR talk*[TIAB] OR help*[TIAB] OR "self-help"[TIAB] OR debriefing[TIAB] OR empathy[TIAB] OR "Social Support"[Mesh] OR "Communication"[Mesh:NoExp] OR "Information Seeking Behavior"[Mesh] OR "Nonverbal Communication"[Mesh] OR "Verbal Behavior"[Mesh] OR "Access to Information"[Mesh] OR "Health Communication"[Mesh] OR "Information Dissemination"[Mesh] OR "Helping Behavior"[Mesh] OR "Empathy"[Mesh] OR "Crisis Intervention"[Mesh]

4. (Resilience[TIAB] OR Efficacy[TIAB] OR Self-efficacy[TIAB] OR Empowerment[TIAB] OR Ability[TIAB] OR Adapt*[TIAB] OR Functioning[TIAB] OR function[TIAB] OR Adjust*[TIAB] OR "Burn out"[TIAB] OR Burnout[TIAB] OR burn-out[TIAB] OR Calm*[TIAB]OR Capacity[TIAB] OR Comfort[TIAB] OR Safety[TIAB] OR comfortable[TIAB] OR Confident[TIAB] OR Connect*[TIAB] OR "Social Contact"[TIAB] OR "Core skills"[TIAB] OR Cope[TIAB] OR coping[TIAB] OR Engagement[TIAB] OR Flexibility[TIAB] OR Hardiness[TIAB] OR Hope[TIAB] OR hoping[TIAB] OR Network*[TIAB] OR "Blood pressure"[TIAB] OR "Heart rate"[TIAB] OR Heartbeat[TIAB] OR "Mental health"[TIAB] OR "Mental Health"[Mesh] OR "Resilience, Psychological"[Mesh] OR "Self Efficacy"[Mesh] OR "Power (Psychology)"[Mesh] OR "Stress, Psychological"[Mesh] OR "Stress Disorders, Traumatic"[Mesh] OR "Sleep Disorders"[Mesh] OR "Substance-Related Disorders"[Mesh] OR "Depressive Disorder"[Mesh:NoExp] OR "Adjustment Disorders"[Mesh] OR "Anxiety Disorders"[Mesh:NoExp] OR "Panic Disorder"[Mesh] OR "Adaptation, Psychological"[Mesh] OR "Trust"[Mesh] OR "Social Behavior"[Mesh:NoExp] OR "Social Adjustment"[Mesh] OR "Social Isolation"[Mesh] OR "Social Networking"[Mesh] OR "Quality of Life"[Mesh] OR "Blood Pressure"[Mesh] OR "Heart Rate"[Mesh] OR "Bereavement"[Mesh] OR PTSD [TIAB] OR ASD [TIAB] OR "Depression"[Mesh] OR "Survival/Psychology"[Mesh])

5. ((("randomized controlled trial"[PT] OR "controlled clinical trial"[PT] OR "clinical trial"[PT] OR "comparative study"[PT] OR random*[TIAB] OR controll*[TIAB] OR "intervention study"[TIAB] OR "experimental study"[TIAB] OR "comparative study"[TIAB] OR trial[TIAB] OR evaluat*[TIAB] OR "Before and after"[TIAB] OR "interrupted time series"[TIAB]) NOT ("animals"[MH] NOT (animals[MH] AND "humans"[MH]))) OR ("Epidemiologic Studies"[Mesh] OR "case control"[TIAB] OR "case-control"[TIAB] OR ((case[TIAB] OR cases[TIAB]) AND (control[TIAB] OR controls[TIAB)) OR “cohort study”[TIAB] OR "cohort analysis"[TIAB] OR "follow up study"[TIAB] OR "follow-up study"[TIAB] OR "observational study"[TIAB] OR "longitudinal"[TIAB] OR "retrospective"[TIAB] OR "cross sectional"[TIAB] OR "cross-sectional"[TIAB] OR questionnaire[TIAB] OR survey[TIAB] OR "Population Surveillance"[Mesh]))

6. AND 1-5

**PsycINFO - experimental and observational studies**

1. (Disaster.ti,ab. OR disasters.ti,ab. OR crisis.ti,ab. OR crises.ti,ab. OR emergency.ti,ab. OR emergencies.ti,ab. OR exp Disasters/ OR terrorism.ti,ab. OR drought*.ti,ab. OR Cyclone*.ti,ab. OR "Cyclonic Storm*".ti,ab. OR hurricane*.ti,ab. OR flood*.ti,ab. OR fire*.ti,ab. OR exp Industrial accidents/ OR "nuclear accident*".ti,ab. OR "tidal wave*".ti,ab. OR "volcanic eruption*".ti,ab. OR tsunami*.ti,ab. OR landslide*.ti,ab. OR avalanche*.ti,ab. OR earthquake*.ti,ab. OR Storm*.ti,ab. OR wildfire*.ti,ab. OR Snowstorm*.ti,ab. OR "heat wave*".ti,ab. OR "cold wave*".ti,ab. OR "land fire*".ti,ab. OR Sandstorm*.ti,ab. OR exp Terrorism/ OR massacre.ti,ab. OR bomb*.ti,ab. OR evacuation.ti,ab. OR exp Motor Traffic Accidents/ OR ((traumatic.ti,ab. OR serious.ti,ab. OR destructive.ti,ab. OR mass.ti,ab. OR stressing.ti,ab. OR critical.ti,ab.) AND (event.ti,ab. OR events.ti,ab. OR incident.ti,ab. OR incidents.ti,ab. OR violence.ti,ab. OR trauma.ti,ab.)))

2. (Provider*.ti,ab. OR responder*.ti,ab. OR worker*.ti,ab. OR helper*.ti,ab. OR professional*.ti,ab. OR volunteer*.ti,ab. OR nurse*.ti,ab. OR EMT.ti,ab. OR "Emergency medical technician*".ti,ab. OR exp Caregivers/ OR laypeople.ti,ab. OR layperson*.ti,ab. OR exp Survivors/ OR evacuees.ti,ab.)

3. (((Psychosocial.ti,ab. OR psychological.ti,ab. OR social.ti,ab OR "mental health".ti,ab.) AND ("first aid".ti,ab. OR care.ti,ab. OR support.ti,ab. OR assist*.ti,ab. OR management.ti,ab.)) OR inform*.ti,ab. OR comforting.ti,ab. OR communicat*.ti,ab. OR conversation.ti,ab. OR talk*.ti,ab. OR help*.ti,ab. OR self-help.ti,ab. OR debriefing.ti,ab. OR empathy.ti,ab. OR exp Social Support/ OR exp Communication/ OR exp Information Seeking/ OR exp Information Dissemination/ OR exp "assistance (social behavior)"/ OR exp Empathy/ OR exp Crisis Intervention/)

4. (Resilience.ti,ab. OR Efficacy.ti,ab. OR Self-efficacy.ti,ab. OR Empowerment.ti,ab. OR Ability.ti,ab. OR Adapt*.ti,ab. OR Functioning.ti,ab. OR function.ti,ab. OR Adjust*.ti,ab. OR "Burn out".ti,ab. OR Burnout.ti,ab. OR burn-out.ti,ab. OR Calm*.ti,ab. OR Capacity.ti,ab. OR Comfort.ti,ab. OR Safety.ti,ab. OR comfortable.ti,ab. OR Confident.ti,ab. OR Connect*.ti,ab. OR "Social Contact".ti,ab. OR "Core skills".ti,ab OR Cope.ti,ab. OR coping.ti,ab. OR Engagement.ti,ab. OR Flexibility.ti,ab. OR Hardiness.ti,ab. OR Hope.ti,ab. OR hoping.ti,ab. OR Network*.ti,ab. OR "Blood pressure".ti,ab. OR "Heart rate".ti,ab OR Heartbeat.ti,ab. OR "Mental health".ti,ab OR exp Mental Health/ OR exp "resilience (psychological)"/ OR exp self efficacy/ OR exp Interpersonal Control/ OR exp Psychological Stress/ OR exp Sleep Disorders/ OR exp Behavior Disorders/ OR exp "Depression (Emotion)"/ OR exp Adjustment Disorders/ OR exp Anxiety Disorders/ OR exp Emotional Adjustment/ OR exp Coping behavior/ OR exp "trust (social behavior)"/ OR Social Behavior/ OR exp Social Adjustment/ OR exp Social Isolation/ OR exp Social Networks/ OR exp "quality of life"/ OR exp Blood Pressure/ OR exp Heart Rate/ OR exp Bereavement/ OR PTSD.ti,ab OR ASD.ti,ab OR exp stress reactions/)

5. AND 1-4

**PILOTS**

- Psychological first aid
- Psychosocial first aid
- (SU.exact("DISASTERS") AND (SU.exact("CRISIS INTERVENTION") OR SU.exact("CRISIS INTERVENTION 03945")))

**The Cochrane Library - experimental and observational studies**

1. ((Disaster*):ti,ab,kw or (“crisis”):ti,ab,kw or (“crises”):ti,ab,kw or (“emergency”):ti,ab,kw or (“emergencies”):ti,ab,kw or (“terrorism”):ti,ab,kw or (drought*):ti,ab,kw or (hurricane*):ti,ab,kw or (Cyclone*):ti,ab,kw or (flood*):ti,ab,kw or (Cyclonic Storm*):ti,ab,kw or (fire*):ti,ab,kw or (nuclear NEXT accident*):ti,ab,kw or (volcanic NEXT eruption*):ti,ab,kw or (tsunami*):ti,ab,kw or (landslide*):ti,ab,kw or (avalanche*):ti,ab,kw or (earthquake*):ti,ab,kw or (Storm*):ti,ab,kw or (wildfire*):ti,ab,kw or (Snowstorm*):ti,ab,kw or ( heat NEXT wave*): ti,ab,kw or (cold NEXT wave*):ti,ab,kw or (land NEXT fire*):ti,ab,kw or (Sandstorm*):ti,ab,kw or (“massacre”):ti,ab,kw or (bomb*):ti,ab,kw or (“evacuation”):ti,ab,kw or [mh “Disasters ”] or [mh “Radioactive Hazard Release”] or [mh “Chemical Hazard Release”] or [mh “Biohazard Release”] or [mh “Terrorism”] or [mh “Accidents, Traffic”] or (((“traumatic”):ti,ab,kw or (“serious”):ti,ab,kw or (“destructive”):ti,ab,kw or (“mass”):ti,ab,kw or (“stressing”):ti,ab,kw or (“critical”):ti,ab,kw) and ((event*):ti,ab,kw OR (incident*):ti,ab,kw or (“violence”):ti,ab,kw or (“trauma”):ti,ab,kw )))

2. ((Provider*):ti,ab,kw or (responder*):ti,ab,kw or (worker*):ti,ab,kw or (helper*):ti,ab,kw or (professional*):ti,ab,kw or (volunteer*):ti,ab,kw or (nurse*):ti,ab,kw or (“EMT”):ti,ab,kw OR (emergency medical technician*):ti,ab,kw or (“laypeople”):ti,ab,kw or (layperson*):ti,ab,kw or (“evacuees”):ti,ab,kw or [mh “Caregivers”] or [mh “Survivors”])

3. ((((“Psychosocial”):ti,ab,kw or (“psychological”):ti,ab,kw or (“social”):ti,ab,kw or ("mental health”):ti,ab,kw) and (("first aid”):ti,ab,kw or (“care”):ti,ab,kw or (“support”):ti,ab,kw or (assist*):ti,ab,kw or (“management”):ti,ab,kw)) or (inform*):ti,ab,kw or (“comforting”):ti,ab,kw or (communicat*):ti,ab,kw or (“conversation”):ti,ab,kw or (talk*):ti,ab,kw or (help* ):ti,ab,kw or (“self-help”):ti,ab,kw or (“debriefing”):ti,ab,kw or (“empathy”):ti,ab,kw or [mh “Social Support”] or [mh “Communication”] or [mh “helping behaviour”] or [mh “Empathy”] or [mh “Crisis Intervention”] OR [mh “Health Communication"] OR [mh “Access to Information”])

4. ((“Resilience”):ti,ab,kw or (“Efficacy”):ti,ab,kw or (“Self-efficacy”):ti,ab,kw or (“Empowerment”):ti,ab,kw or (“Ability”):ti,ab,kw or (Adapt*):ti,ab,kw or (“Functioning”):ti,ab,kw or (“function”):ti,ab,kw or (Adjust*):ti,ab,kw or (“Burn out”):ti,ab,kw or (“Burnout”):ti,ab,kw or (“burn-out”):ti,ab,kw or (Calm*):ti,ab,kw or (“Capacity”):ti,ab,kw or (“Comfort”):ti,ab,kw or (“Safety”):ti,ab,kw or (“comfortable”):ti,ab,kw or (“Confident”):ti,ab,kw or (Connect*):ti,ab,kw or ("Social Contact”):ti,ab,kw or ("Core skills”):ti,ab,kw or (“Cope”):ti,ab,kw or (“coping”):ti,ab,kw or (“Engagement”):ti,ab,kw or (“Flexibility”):ti,ab,kw or (“Hardiness”):ti,ab,kw or (“Hope”):ti,ab,kw or (“hoping”):ti,ab,kw or (Network*):ti,ab,kw or ("Blood pressure”):ti,ab,kw or ("Heart rate”):ti,ab,kw or (“Heartbeat”):ti,ab,kw or ("Mental health”):ti,ab,kw or [mh ”Mental Health”] or [mh “self efficacy”] or [mh “Resilience, Psychological”] or [mh “Stress, Psychological”] or [mh “Sleep Disorders”] or [mh “Stress Disorders, Traumatic”] or [mh “Depression Emotion"] or [mh “Adjustment Disorders”] or [mh “Adaptation, Psychological”] or [mh “trust”] or [mh “Power (Psychology)”] or [mh “Social Adjustment”] or [mh “Social Isolation”] or [mh “Panic Disorder”] or [mh “Substance-Related Disorders”] or [mh ^“Depressive disorder”] or [mh “quality of life"] or [mh “Blood Pressure”] or [mh “Heart Rate”] or [mh “Bereavement”] or (“PTSD”):ti,ab,kw or (“ASD”):ti,ab,kw or [mh “Survival”])

5. AND 1-4
