# Supplementary material for: A Systematic Literature Search on Psychological First Aid: Lack of Evidence to Develop Guidelines
Source: PLoS One. 2014 Dec 12;9(12):e114714. doi: 10.1371/journal.pone.0114714 (PMC4264843; doi:10.1371/journal.pone.0114714)
Supplement: S2 Appendix — Excluded studies after full text evaluation. (DOC) [file pone.0114714.s003.doc]

**APPENDIX S2.** List of excluded studies after full text evaluation.

List of excluded studies: Systematic literature search

List of excluded studies (continued)

List of excluded studies (continued)

List of excluded studies (continued)

List of excluded studies (continued)

List of excluded studies : Studies from TENTS guidelines

List of excluded studies : Studies from EUTOPA guidelines and Te Brake et al.
